# Sex-for-Crack exchanges: associations with risky sexual and drug use niches in an urban Canadian city

**DOI:** 10.1186/1477-7517-10-29

**Published:** 2013-11-15

**Authors:** Putu Duff, Mark Tyndall, Jane Buxton, Ruth Zhang, Thomas Kerr, Kate Shannon

**Affiliations:** 1 British Columbia Centre for Excellence in HIV/AIDS, St. Paul’s Hospital, 608-1081 Burrard Street, Vancouver, BC V6Z 1Y6, CANADA; 2 School of Population and Public Health, University of British Columbia, 2206 East Mall, Vancouver, BC V6T 1Z3, CANADA; 3 Department of Medicine, General Campus, University of Ottawa, The Ottawa Hospital, 501 Smyth Road, Box 206, Ottawa, ON K1H 8 L6, CANADA; 4 British Columbia Centre for Disease Control, 655 West 12th Avenue, Vancouver, British Columbia V5Z 4R4, CANADA; 5 Department of Medicine, University of British Columbia, St. Paul’s Hospital, 608-1081 Burrard Street, Vancouver, BC V6Z 1Y6, CANADA

**Keywords:** Crack cocaine, Sex work, Sexual risk, HIV, STI

## Abstract

**Background:**

While crack cocaine has been associated with elevated sexual risks and transmission of HIV/STIs, particularly in the context of street-based sex work, few empirical studies have examined correlates of direct sex-for-crack exchanges. This study longitudinally examined the correlates of sex-for-crack exchanges and associated effects on sexual risk outcomes among street-based female sex workers (SW) who use drugs in Vancouver, Canada.

**Methods:**

Data were drawn from a prospective cohort of street-based SWs (2006–2008), restricted to those who smoke crack cocaine. Multivariable generalized estimating equations (GEE) were employed to examine the correlates of exchanging sex for crack. A confounding model using GEE quasi-Poisson regression modeled the independent effect of exchanging sex for crack on number of clients/week.

**Results:**

Of 206 SWs, 101 (49%) reported sex-for-crack exchanges over 18 months of follow-up. In multivariable GEE analyses, sharing a crack pipe with a client (aOR = 1.98; 95%CI: 1.27-3.08) and smoking crack in a group of strangers (e.g., in an alley or crackhouse) (aOR = 1.70; 95% CI: 1.13-2.58) were independently correlated with sex-for-crack exchanges. In our confounding model, exchanging sex for crack (aIRR = 1.34; 95% CI: 1.07-1.69) remained significantly associated with servicing a greater number (>10) of clients/week.

**Conclusions:**

These findings reveal elevated sexual- and drug- risk patterns among those who exchange sex for crack. The physical and social environment featured prominently in our results as a driver of sex-for-crack exchanges, highlighting the need for gender-sensitive multilevel approaches to harm reduction, STI and HIV prevention that address SWs’ environment, individual level factors, and the interplay between them.

## Introduction

The advent of widespread use of crack cocaine in North America in the 1980s and 1990s has been directly linked to elevated rates for sexually transmitted infections (STIs), including HIV transmission [1-3], through increased sexual risk pathways (e.g. higher number of sexual partners and unprotected sex) [4,5]. Crack cocaine use has been documented as a predictor for both HIV and HCV, even after adjusting for known confounders such as injection drug use, suggesting a non-parenteral risk pathway [1,6]. For example, a Canadian study found that crack smoking was associated with a 4.01 increased odds of HIV acquisition, while an American study reported a 3.87 increased odds of acquiring HCV among participants who used crack [1,6]. In addition to the sexual and drug risk pathways, the use of non-injection crack cocaine has been linked to an array of adverse physical and mental health outcomes, including elevated individual and community-level violence and physical health harms, such as oral sores and pulmonary complications [5,7,8].

While the population prevalence of crack use varies across settings, a growing number of studies in high-income settings have suggested that street-involved women’s crack use may exceed men’s [9-11]. For example, among a sample of treatment-seeking individuals who use drugs, Lejuez and colleagues reported that 84.5% of women reported crack use compared to 63.6% of men. In Florida, a study among drug-using inmates found that 74% of females reported crack as their primary drug, compared to 49% of men [12]. Similar trends have also been reported in the Vancouver context; the reported prevalence of daily crack use among street-involved women was found to be 9.7% compared to 5.6% among men [1]. Though it is unclear whether the higher prevalence among street-involved women is related to sex work, studies elsewhere have noted high levels of crack cocaine among sex workers [5,13]. Qualitative researchers in our settings have highlighted a need to better understand the contextual factors that drive crack use and associated outcomes [11]. STIs and other health risks posed by crack use are exacerbated by the environmental and social contexts such as interpersonal-violence, poverty, homelessness, incarceration and stigmatization that street-based SWs often contend with [13]. For example, street-based SWs who use illicit drugs, including crack, have reported limited access to health and harm reduction services due to avoidance of policing and client violence [14].

Despite growing evidence linking street-based sex work and crack use [15,16], as well as the numerous harms associated with using the drug, the epidemiological understanding of the individual and contextual correlates of “sex-for-crack exchanges” and associated sexual risk outcomes among street-based SWs is limited. While existing STI and HIV research have focused primarily on drug-related harms [17], and to a lesser extent sexual-related risks, few have explicitly examined risks and outcomes among street-based SWs who exchange sex for crack. Furthermore, most existing epidemiological studies related to crack use among women have focused on individual-level factors, such health behaviors, drug use, ethnicity, gender and age. While these characteristics are important, they do not fully capture factors more distal to the individual such as the larger context of crack use, including sex-for-crack exchanges, gender-relations, interpersonal violence, as well as other environmental-level factors that drive and shape crack use [11]. This study therefore sought to longitudinally examine the individual-, interpersonal- and environmental-level correlates and outcomes of exchanging sex-for-crack among a population of street-based SWs in Vancouver, Canada.

## Methods

This study was a secondary analysis drawn on data from a community-based prospective cohort, partnered with local sex work and community service agencies, and has been described in detail previously [18]. Briefly, between 2006–2008, 252 women (inclusive of transgendered individuals) engaged in street-based sex work were recruited through outreach and participated in an informed consent process. The response rate of SWs contacted for interview was 94%. Time-location sampling and mapping by peer research team (current/former sex workers) were used to identify sex work spaces for targeted outreach and recruitment. Eligibility for the study included being female (inclusive of transgender male-to-female), 14 years of age or older, having exchanged sex for money or resources in the last 30 days, and have used illicit drugs (other than cannabis). Baseline and 6-monthly follow-up surveys were completed by participants, and consisted of interview-administered questionnaires by a peer researcher (current/former street-based sex worker), nurse-administered pre-test counseling questionnaire, and HIV screening. A $25 honoraria was provided to respondents at each 6-monthly visit as compensation for their time and expertise. Ethical approval was provided by UBC/Providence Health ethics review board.

### Main outcome measure

The primary outcome for this study was having responded 'yes’ to having 'exchanged sex directly for your next rock (crack cocaine)’ in the previous 6 months. Based on existing literature and *a priori* knowledge of sexual risks of crack smokers, we developed a separate confounding model to examine the independent effect of “exchanging sex directly for crack” (main explanatory variable) on number of clients/week (continuous).

### Explanatory variables

Based on the literature, individual, interpersonal/social environment and physical environmental factors were selected as explanatory variables in our analyses. Individual factors included socio-demographic factors (e.g., age, education) and drug use patterns (any use of cocaine, heroin, crack cocaine, crystal methamphetamine in the last 6 months). In Vancouver, people of Aboriginal ancestry are overrepresented in street-based sex work [19], and are disproportionately affected by socioeconomic inequities such as poverty, homelessness and substance use [20,21]. Given the overrepresentation of individuals of Aboriginal ancestry (inclusive of First Nations, Metis, Inuit ancestry) in street-based SW and drug use populations in Canada, we adjusted for Aboriginal ancestry vs. non-Aboriginal ancestry (Caucasian). Due to the limited number of participants from other ethnic backgrounds among our sample, we did not adjust/stratify by any other ethnic groups. Given high rates of daily crack cocaine use among SWs, we stratified crack cocaine smoking at the mean by intensive (≥10 rocks/day) vs. less intensive use (<10 rocks/day) [18]. Interpersonal and social environmental variables considered in our analyses included: using drugs with a regular client, borrowing used rigs, smoking crack cocaine in a group of strangers (e.g., in crack houses or alleys), sharing drugs with clients, servicing a higher number of clients (stratified at the mean, >10), inconsistent condom use for vaginal sex by clients (defined as 'always’ versus 'usually’ , 'sometimes’ , 'occasionally’ or 'never’ , client perpetrated violence (bad dates), and physical or sexual violence by intimate partners. Economic dependence on one’s partner (defined as “having a partner who scores drugs for you”, and having a regular partner that supports you financially) was also considered as interpersonal risk factors for our analyses. Physical environmental factors considered were: smoking crack in public spaces; homelessness (defined as sleeping on the street); police affecting where you get drug equipment; servicing clients in outdoor/indoor spaces; and working in main/commercial areas or side-streets/alleyways/industrial areas.

### Statistical analysis

The sample was restricted to 206 SWs who smoked crack in the last 6 months and completed baseline and at least one follow-up visit. As it was an open cohort study with staggered enrolment, all participants had at least 6 months of observation, with a few having 12 and 18 months of observation (for a maximum of 4 time points). Descriptive statistics, including frequencies, proportions, medians, interquartile ranges [IQR] were provided for baseline individual, interpersonal and environmental factors and were stratified by whether or not the participant had exchanged sex for crack within the past 6 months. Baseline and follow-up data capturing socio-demographic characteristics (e.g., ethnicity, education) were treated as fixed covariates, and all other variables (e.g., age, housing and drug use status) were treated as time-updated covariates of occurrences within the past six months of the interview. Bivariate and multivariable logistic regression using Generalized Estimating Equations (GEEs) were conducted and included information from each participant’s baseline and follow-up questionnaires. We used generalized estimating equations (GEE) with a logit link for our binary outcome to take into account correlations arising from repeated measures on the same individuals over the follow-up period (this also accounted for varying observations lengths between participants). Standard errors adjusted by repeated observations per person were obtained using an exchangeable correlation structure. Missing data and intermittent data were handled using the GEE estimating mechanism, which draws on data from non-missing pairs for the estimators of its working correlation matrix. Variables significantly associated with exchanging sex for crack in the bivariate analyses at the p < 0.10 level were subsequently fitted in a multivariable GEE model. Two-sided p-values, bivariate and adjusted odds ratios (OR and aOR) with 95% confidence intervals (95% CI) were reported.

To determine independent associations with exchanging sex for crack, bivariate screening of *a priori* and hypothesized confounders was conducted, with variables associated with exchanging sex for crack at p < 0.10 considered for inclusion in the multivariable explanatory model. Akaike Information Criteria (AIC) selection was used to arrive at the final multivariable model. The final model was assessed for multicollinearity. To assess if exchanging sex for crack was independently associated with number of clients per week, a confounding model was constructed using an approach described by Rothman and Greenland [22]. Confounders were chosen based on *a priori* knowledge of associations with sex-for-crack-exchanges *and* number of commercial partners. These potential confounders underwent bivariate screening, and those that retained significance at p < 0.10 were considered potential confounders and were included in the multivariable confounding model. As in previous studies [23,24], all potential confounders were included in a full model, and subjected to a manual stepwise approach, where variables that altered the association of interest by less than 10% were systematically removed from the model. As in a previous analysis [24], age was forced into the multivariable confounding model and not subjected to the manual stepwise approach due to the well-established confounding effects of this variable. SAS statistical software package version 9.2 was used for all data analyses (SAS Institute, Cary, NC, USA).

## Results

Of a total of 252 participants enrolled in our open prospective cohort between 2006–2008, 206 (82%) had reported smoking crack within the follow-up period. As in Table 1, 101 (49%) reported exchanging sex for crack, and the median age of participants who exchanged sex for crack was 35 years [IQR: 25.0-40.0] compared to 37 years [IQR: 25.0-40.0] among those who did not report having exchanged sex for crack-cocaine, with just under half (48.5%) who were of Aboriginal ancestry (inclusive of First Nations, Metis, Inuit ancestry and non-status First Nations). The median age of initiation into crack use was slightly younger (20 years; IQR: 16–27) among those who exchanged sex for crack compared to those who did not (21 years; IQR:16–30).

**Table 1:** Socio-demographic, interpersonal/social environment and physical-environment characteristics of sex-for- crack exchanges among a cohort of street-based female SWs in Vancouver, Canada

**Characteristic**	**Total (%)**	**Sex-for-crack exchanges**		***p *- value**
		**Yes ( *% *)**	**No (%)**	
	**(*n*** **= 206)**	**(*n*** **= 101)**	**(*n*** **= 105)**	
**Socio-demographic**				
Age (med, IQR)		35 (25–40)	37 (28–41)	0.091
Aboriginal ancestry	100 (48.5)	57 (57.00)	43 (43.00)	0.175
Caucasian	106 (51.5)	48 (45.28)	58 (54.72)	–
Age first used crack (median, IQR)		20 (16–27)	21 (16–30)	–
**Individual level- *drug risks***				
Cocaine injection*	73 (35.40)	46 (22.30)	27 (13.11)	0.050
Heroin injection*	104 (50.49)	62 (59.62)	42 (40.38)	0.043
Crystal Meth injection/non-injection*	24 (11.65)	6 (25.00)	18 (75.00)	0.186
Intensive crack use (>10 rocks/day)*	57 (27.67)	25 (43.86)	32 (56.14)	0.021
**Interpersonal/ social environment**				
Used drug with regular client*	72 (35.00)	41 (56.94)	31 (43.06)	0.110
Receptive sharing of used syringe *	16 (7.77)	10 (62.50)	6 (37.50)	0.003
Shared used pipe with regular client/john*	95 (46.12)	59 (62.11)	36 (37.89)	<0.001
Higher number of clients (>10)*	71 (37.60)	42 (22.20)	29 (15.34)	<0.001
Inconsistent condom use with client (for vaginal sex)*	19 (9.22)	12 (63.16)	7 (36.84)	0.031
Intimate partner uses drugs	74 (35.92)	38 (51.35)	36 (48.65)	0.352
Intimate partner provides drugs	55 (26.70)	30 (54.55)	25 (45.45)	0.260
Economic dependence on intimate partner	11 (5.34)	6 (54.55)	5 (45.45)	0.490
Physical/sexual violence by client*	45 (21.84)	29 (64.44)	16 (35.56)	0.002
**Physical environment**				
Smoke crack in groups with strangers (e.g., crack houses, alleys)*	165 (80.10)	89 (53.94)	76 (46. 06)	0.001
Homeless*	88 (42.7)	50 (56.82	38 (43.18)	<0.003
Work in alleyways, industrial areas*	134 (65.00)	69 (51.49)	65 (48.51)	<0.001
Services clients in public spaces*	141 (68.45)	74 (52.48)	67 (47.52)	0.001

*In the last 6 months.

The results of bivariate and multivariable GEE model are presented in Table 2. In our multivariable GEE explanatory model, sharing a crack pipe with a regular or one-time client [aOR = 1.98; 95% CI: 1.27-3.09] and smoking crack with a group of strangers [aOR = 1.70; 95% CI: 1.13-2.58] remained significantly correlated with exchanging sex-for-crack.

**Table 2:** Bivariate and multivariable logistic regression models using generalized estimating equations (GEEs) for correlates of sex-for-crack exchanges among street-involved sex workers in Vancouver, Canada

**Characteristic**	**Unadjusted**		**Adjusted**	
	**Odds ratio**	***p - *** **value**	**Odds ratio**	***p - *** **value**
	**(95% CI)**		**(95% CI)**	
**Socio-demographic**				
Age^‡^	0.98 (0.96 –1.00)	0.091	0.99 (0.96–1.01)	0.230
Aboriginal ancestry vs. Caucasian	0.74 (0.48 –1.14)	0.175	–	–
**Individual level- *drug risks***				
Cocaine Injection*	1.52 (1.00 – 2.31)	0.050	1.29 (0.82 –2.03)	0.275
Heroin Injection*	1.57 (1.01 – 2.42)	0.043	1.12 (0.69 –1.82)	0.653
Intensive crack use*†	1.71 (1.09 – 2.69)	0.021	–	–
Crystal meth injection/non- injection	0.61 (0.29 – 1.27)	0.186	–	–
**Interpersonal/ social environment**				
Shared used pipe with regular client/john*	2.31 (1.54 – 3.47)	<0.001	1.93 (1.28–2.91)	0.002
Intimate partner uses drugs†	1.23 (0.80 – 1.87)	0.352	–	–
Intimate partner provides drugs†	1.31 (0.82 – 2.08)	0.260	–	–
Physical/sexual violence by client*†	2.27 (1.37 – 3.78)	0.002	–	–
Inconsistent condom use by client (for vaginal sex)†*	2.25 (1.08 – 4.70)	0.031	–	–
Serviced over 10 clients/week*†	2.18 (1.39 – 3.43)	<0.001	–	–
**Physical environment**				
Smoke crack in groups with strangers e.g., crack houses, alleys*	2.14 (1.44 – 3.17)	0.001	1.70 (1.13–2.58)	0.012
Homeless*†	1.91 (1.25 – 2.93)	<0.003	–	–
Work in alleys/industrial areas†*	2.30 (1.53 – 3.46)	0.001	–	–
Services clients in public spaces*†	2.03 (1.32 – 3.13)	0.001	–	–

*Last 6 months.

†Variable not entered into logistic model.

^‡^Age was forced into the model based on *a priori* knowledge as a confounder.

The results of our confounding model examining the independent effect of exchanging sex for crack on number of clients/week are shown in Table 3. In our confounding model (adjusting for age, servicing clients in public spaces, police affecting access to drug equipment), having exchanged sex for crack in the past six months was associated with a 34% increased risk of greater than average number of clients (>10 clients/week) (Incidence Rate Ratio (IRR): 1.34 [95% Confidence Interval 1.07- 1.69]). We also constructed a multivariable confounding model for the relationship between exchanging sex for crack and inconsistent condom use, which yielded statistically non-significant results.

**Table 3:** Multivariable confounding model of the independent effect of sex-for-crack exchanges on number of clients among a cohort of street-based female sex workers in Vancouver, Canada

**Characteristic**	**Unadjusted incidence rate ratio**		**Adjusted incidence rate ratio**	
	**IRR (95% CI)**	***p - *** **value**	**aIRR (95% CI)**	***p - *** **value**
Exchanged sex for crack*	1.55 (1.22– 1.97)	<0.001	1.34 (1.07- 1.69)	0.013

^*^Within the last 6 months.

N.B. Multivariable confounder model adjusted for *a priori* and statistically significant confounders (servicing clients in public spaces, and age). Age was forced into the model based on well-established *a priori* knowledge of age as a confounder.

## Discussion

The results of this study demonstrate that among street-based sex workers who smoke crack, a large proportion (49%) reported non-monetary, direct sex-for-crack exchanges. These findings highlight the importance of intersecting social and physical contexts in driving sex-for-crack exchanges and sexual and drug risks among street-based SWs. These results provide epidemiological data to confirm qualitative reports among drug users in our setting that suggest marginalized physical spaces, such as alleys, are 'niche’ settings for illicit drug use, where using in groups, and sharing drug-paraphernalia is the norm [25]. While people who use drugs have described these 'niches’ as far from an ideal setting for drug use [25], it is important to acknowledge that they are a by-product of a number of structural factors including homelessness, lack of access to safe spaces to smoke, stigma associated with drug-use and sex work, and avoidance of law enforcement [11]. Crack cocaine is a common feature of the street economy, with crack use 'niches’ (e.g., alleys or crack houses) often concentrated around sex work strolls, social housing and vacant lots; spaces where some of the most marginalized and stigmatized populations live and congregate [26].

The physical features of drug using 'niches’ may act as a 'site of social and cultural reproduction’ [27], where the dynamic interplay between individual-level factors and their environments foster and perpetuate sex-for-crack exchanges. Ethnographic accounts describe how the addictive and stigmatized nature of crack creates a cycle of use that quickly deteriorates street-based SWs’ work environments and becomes entrenched as a central feature of street-based sex work [27]. Street-based SWs’ exposure to high-risk environments such as smoking in groups of strangers in isolated, unsanitary public spaces such as alleys or crack houses may facilitate the creation of social ties with other drug users, intensive daily crack use and sharing of paraphernalia, that has been posited to link crack use and STI transmission [1,6]. Furthermore, 'niches’ such as alleys and crack houses often reinforce a culture where sex-for-crack exchanges represent a highly gendered power dynamic. For example, sex-for-crack exchanges in these settings often occur in the context of intense cravings/withdrawals that may facilitate sex-for-crack exchanges while high, and exacerbate female SWs’ vulnerability to gender-based violence, STI and HIV transmission, including through reduced ability to negotiate for condom use by clients and clients insisting on sex without a condom [28]. Though crack cocaine use has been associated with gender-based violence and inconsistent condom use elsewhere [29], we did not find a statistically significant association between either client violence or inconsistent condom use and exchanging sex for crack, after adjusting for potential confounders. This findings is somewhat surprising, given qualitative accounts from women who use drugs in our setting that indicate that smoking crack, particularly in unsafe areas such as alleys (due to displacement from homelessness, policing, lack of safe smoking places) often increases the risk of gender-based violence [11]. The exclusive focus on crack-using sex workers (versus a sample of drug-using women) in our sample may have contributed to the lack of association. The lack of association with violence may reflect the general pervasiveness of violence among women who use crack, (regardless of whether or not they exchange sex for crack), resulting in a similar distribution of violence between those who engage in sex-for-crack exchanges and those who do not.

While two decades of qualitative and ethnographic work have described the physical and social contexts of crack houses featured prominently in street-based SW’s sex-for-crack exchanges [30], this is among the first studies to longitudinally examine the social and physical features independently linked with sex-for-crack exchanges. In Inciardi et al.’s study, SWs working primarily in crack houses reported an association between exchanging sex for crack and higher number of clients [17]. While some studies make clear distinctions between street-based vs. crack house sex workers, others do not. Inciardi (1995) makes a clear distinction between SWs who work on the street and those who work in crack houses, describing crack house-based SWs as highly addicted, desperate and reliant on crack-house pimps/managers, often accepting the lowest price for a hit of crack or exchanging sex for a smoke [31]. The high drug-dependency among SWs who work in crack houses, paired with low pay and high traffic in these settings are described to contribute to the higher number of clients among street-based SWs who work in crack houses [31]. In contrast, another qualitative study described sex-for-crack exchanges as occurring in the same physical settings as sex-for-cash transactions, and did not observe a clear distinction between those who engage in sex-for-crack exchanges and those who do not [27]. While sex-for-crack exchanges were considered degrading and purposefully avoided by many street based SWs, Maher’s ethnographic research suggests that these transactions occur under pressing circumstances, where women considered the need for crack to outweigh the shame of exchanging sex to obtain the drug [27]. In our study, smoking in anonymous groups (both crack houses and alleys) was independently correlated with increased likelihood of exchanging sex directly for crack. The greater number of clients (>10 per week) reported by street-based SWs who exchange sex for crack in our sample likely reflects women’s need to support their intense crack use patterns [32], and underlines the vicious cycle of sex work and addictions driving these sex-for-crack exchanges.

This study also revealed increased odds of reciprocal crack-pipe sharing with clients, and smoking crack in groups with strangers (e.g., in crack houses or alleys) among SWs’ who exchanged sex for crack, after adjusting for potential confounders in multivariable analysis. Our confounding model indicates that exchanging sex for crack was associated with an increased number of clients, after controlling for potential confounders. The increased odds of sex-for-crack exchanges among street-based SWs who smoke crack with groups of strangers (namely, alleys or crack houses), and share crack pipes (reciprocally) with clients underscores the importance of social and structural environments in shaping sex-for-crack exchanges and STI transmission.

These findings support structural and environmental interventions, such as the removal of criminal sanctions that would enable SWs to work (and smoke) in safer indoor spaces (including low-barrier housing supports and/or workspaces). Such interventions would remove SWs’ need to smoke in public outdoor spaces with strangers and/or clients, where sharing of smoking paraphernalia is common. Such indoor settings may alter gendered-power dynamics of sexual exchanges, potentially improving SWs’ ability to negotiate for higher rates per transaction, thus reducing the need to service a greater volume of clients. Increased access to safer smoking kits may improve SWs’ choices related to where and with whom they smoke [30]. Another alternative is the implementation of safer smoking facilities (SSFs), particularly within close proximity to SWs’ workspaces. SSFs may increase access to clean crack pipes, reduce the risk of pipe sharing with clients and provide an environment for safer smoking practices. As well, SSFs may increase exposure to health care and addiction treatment services, reduce public smoking and move street-based SWs who exchange sex for crack away from alleys and crack houses. The development and evaluation of SSFs that specifically cater to the needs of SWs may also be beneficial, and could further moderate violence and coercion in drug and sex work scenes. Further research is needed to identify acceptable and effective models for crack use harm reduction. Finally, gender-specific programs targeting women who exchange sex for crack should be developed that address the gendered-power dynamics present in sex-for-crack exchanges.

This study has a number of limitations that should be noted. The findings from this study may not be generalizable to SWs working in other venues, such as bars, massage parlours and/or escort agencies. However, this limitation is tempered by our time-location sampling method, which is used to recruit hard-to-reach populations by sampling at times and places where they are known to congregate. Social mapping of spaces of servicing and solicitation were conducted beforehand with current and former SWs. While we employed statistical methods commonly used to analyze data collected using time-location sampling, emerging evidence suggests there are other methods that may better account for clustering by sampling location and variability in the probability of sampling among members (i.e., treating the sample as a two-staged sample) [33]. As a result, our statistical methods may have underestimated the true standard errors, as well as affected the estimates of interest. Also, though causality cannot be inferred from this study, due to the observational nature of the research, some potential temporal bias may be reduced due to the use of generalized estimating equations (GEEs) that account for repeated measurements on the same respondents. This study used self-report data, and women’s responses may be subject to social desirability bias (i.e. when women report answers they view as being socially acceptable to the interviewer or society). However, a number of studies have found SWs and drug users to provide truthful accounts of their sex and drug use activities when questioned in a non-threatening environment [34], and we believe the community-based nature of our study serves to reduce the likelihood of this form of response bias. Questions pertaining to events that occurred within the past 6 months of the interview may be subject to recall bias. The arbitrary 6 month cut-off may also result in an underestimation of our estimates, as sex-for-crack exchanges occurring more than 6 months prior to the interview would go unreported. Finally, while the data collection for this study began in 2005, our continued research in this setting (among street- and off-street sex workers) indicates these drug-use 'niches’ and sex-for-crack exchanges persist, and these findings remain a relevant and important issue for this population.

These findings reveal that sex-for-crack exchanges by street-based SWs may increase risk for STI and HIV transmission, through the sharing of crack pipes with clients and servicing a higher volume of client (if in the context of inconsistent condom use). The physical and social environments may be important drivers of sex-for-crack exchanges, highlighting an urgent need for multilevel approaches to harm reduction, STI and HIV prevention that address street-based SWs’ environment, individual level factors, and the interplay between them. These findings reveal that sex-for-crack exchanges by street-based SWs may increase risk for STI and HIV transmission, through the sharing of crack pipes with clients and servicing a higher volume of client (if in the context of inconsistent condom use). The physical and social environments may be important drivers of sex-for-crack exchanges, highlighting an urgent need for multilevel approaches to harm reduction, STI and HIV prevention that address street-based SWs’ environment, individual level factors, and the interplay between them. These findings point to a need for alternative models for crack use harm reduction that are gender sensitive, and serve the needs of sex workers who exchange sex for crack. In the interim, there is a need to improve access to clean drug use equipment (e.g., through safer smoking facilities and the distribution of safer smoking kits). Additionally, the removal of criminal sanctions that prevent SWs from working (and smoking) in safer indoor spaces may help reduce sex-for-crack exchanges among this population.

## Competing interests

The authors declare that they have no competing interests.

## Authors’ contributions

KS had access to the data and takes full responsibility for the integrity of the data. PD and KS developed the analyses plan, and RZ conducted the statistical analyses. PD wrote the first draft of the manuscript and integrated suggestions from all co-authors. All authors made significant contributions to the conception and design of the analyses, interpretation of the data, and drafting of the manuscript, and all authors approved the final manuscript.
